# Truncated Bovine Integrin Alpha-v/Beta-6 as a Universal Capture Ligand for FMD Diagnosis

**DOI:** 10.1371/journal.pone.0160696

**Published:** 2016-08-05

**Authors:** Gareth Shimmon, Britta A. Wood, Alison Morris, Valerie Mioulet, Santina Grazioli, Emiliana Brocchi, Stephen Berryman, Toby Tuthill, Donald P. King, Alison Burman, Terry Jackson

**Affiliations:** 1 The Pirbright Institute, Ash Rd, Pirbright, Surrey, GU24 0NF, United Kingdom; 2 Istituto Zooprofilattico Sperimentale della Lombardia e dell'Emilia Romagna, Via Bianchi 9, Brescia, Italy; Saint Louis University, UNITED STATES

## Abstract

Foot-and-mouth disease (FMD) is endemic in many regions of the world and is one of the most prevalent epizootic animal diseases. FMD affects livestock, such as cattle, sheep, goats and pigs, and causes enormous economic losses due to reduced productivity and trade restrictions. Preparedness and early diagnosis are essential for effective control of FMD. Many diagnostic assays are dependent on raising high-affinity, anti-FMD virus (FMDV) serotype-specific antibodies in small animals (rabbits and guinea pigs) that give broad virus coverage. Here we show that soluble, truncated forms of bovine αvβ6 bind FMDV in an authentic RGD and divalent cation dependent interaction and can be used as the trapping reagent in a FMDV sandwich ELISA. In addition, inclusion of FLAG or His tags facilitates simple purification without the loss of virus binding. We also provide evidence that when combined with a guinea pig polyclonal serum, or serotype-specific monoclonal antibodies, the integrin can be used to detect viruses representative of all FMDV serotypes. We also show that recombinant FMDV empty capsids, with stabilising disulphide bonds, can serve as an antigen in the ELISA and can therefore replace inactivated virus antigen as a positive control for the assay. Our results demonstrate the potential use of bovine αvβ6 and FMDV empty capsids in FMD diagnostic assays.

## Introduction

Foot-and-mouth disease (FMD), caused by FMD virus (FMDV), is endemic in many regions of the world, and is one of the most prevalent epizootic animal diseases [1]. FMD is feared because it is highly contagious and causes enormous economic losses due to reduced productivity and trade restrictions on animals and animal products imposed on affected countries [1]. In 2012 more than 100 countries were recognised as not free of disease by the World Organisation for Animal Health (OIE). FMD affects a large number of animals globally, including domesticated livestock (e.g. cattle, sheep, goats and pigs) and wild animals, which greatly complicate control measures [2]. In addition, FMDV exists as seven serotypes (O, A, C, Asia-1 and Southern African Territories [SAT] SAT-1, SAT-2 and SAT-3) and each serotype is formed by multiple, constantly evolving strains, which further complicates control [3, 4]. Surveillance and early detection of FMD are the cornerstones of successful control strategies and are essential for countries that do not use routine vaccination as a control measure. A sandwich antigen-detection ELISA is routinely used for FMD diagnosis and virus serotyping; these assays require serotype-specific polyclonal sera produced in rabbits and guinea pigs that are used to trap antigen and as the primary detecting antibody respectively [5, 6]. Hence, important limitations of this assay are the need to consistently generate high-affinity, serotype-specific antisera and the need to ensure coverage of new emerging strains.

All field isolates of FMDV use a number of arginine-glycine-aspartic acid (RGD)-binding integrins as cell receptors to initiate infection [7–10]. Integrin binding is mediated by a conserved RGD motif that is located on an exposed loop on the outer surface of the capsid (the G-H loop of VP1) [11]. Integrins are a family of cell surface adhesion receptors that bind to both soluble ligands and ligands that reside within the extracellular matrix. Integrins are heterodimers formed by the non-covalent association of two subunits (α and β). Each subunit has an ectodomain, single transmembrane region and a cytoplasmic domain. The ectodomains from the α and β subunits associate to form the ligand binding site [12].

Previously we demonstrated that FMDV is highly adapted to use one such integrin, αvβ6 [13–16], and that a truncated (lacking the transmembrane and cytoplasmic domains), soluble human αvβ6 (purified from a CHO cell line stably expressing αvβ6) can be used to replace the rabbit polyclonal antibodies as the virus trapping reagent in the standard FMDV sandwich ELISA [17, 18]. Here we describe the generation and characterisation of recombinant, truncated, bovine αvβ6 by transient transfection of HEK293T cells and its potential use in FMDV diagnostic assays. We also show that recombinant FMDV empty capsids (EC) can be used as a positive control antigen in place of inactivated virus preparations.

## Materials and Methods

### Production of empty capsids

The vaccinia virus expression system for producing covalent stabilised FMDV A22 Iraq EC has been previously described [19]. To express the stabilised EC with KGA, the RGD sequence of the MluI to NotI fragment of plasmid pBG200-A22-H2093C [19] was replaced by KGA and the fragment synthesized de novo by GeneArt (Invitrogen/ Thermo Fisher Scientific). Vaccinia virus recombinants were then generated and purified on a 15–45% sucrose gradient as described previously [19].

### Construction of integrin expression plasmids

The sequences of the bovine αv and β6 subunits (including the signal sequences) used in this study were the NCBI reference sequences, NM-174367.1 and NM-174698.2, respectively. Synthetic DNA for the αv and β6 subunits were generated by GeneArt and extended from the ATG translation start codon at the beginning of the signal peptide to the codon for valine at residue 991 (αv) and isoleucine at residue 708 (β6). For both subunits, the 5’ end was extended to include a Kozak sequence before the ATG and the 3’ end was extended to include 2 translation stop codons. For both integrin subunits, the 5’ and 3’ ends were further extended to include unique NheI (5’) and NotI (3’) restriction enzyme recognition sites to facilitate cloning. For the β6 subunit, a unique AgeI restriction enzyme recognition site was introduced during DNA synthesis; this was done by including 2 silent mutations at the coding sequence for a threonine/glycine pair at residues 601–602 (aca-gga to acc-ggt). The appropriately digested fragments were ligated in to similarly digested pCDNA 3.1(+) (Invitrogen/ Thermo Fisher Scientific) to create one plasmid for each subunit. The FLAG or 6xHis tag was added to the C-terminus of the β6 subunit using an AgeI-NotI fragment (GeneArt: Invitrogen/ Thermo Fisher Scientific) corresponding to the original synthesised sequence, but including extra sequence to introduce a FLAG or His tag immediately before the stop codons. The 6xHis tag was added to the C-terminus of the αv subunit using the NotI site and a unique EcoR1 site toward the 3’ end (nt 2874) of the αv subunit. The sequence of the EcoRI-NotI fragment (GeneArt: Invitrogen/ Thermo Fisher Scientific) corresponded to the original sequence but included extra sequence to introduce six histidine residues immediately before the stop codons.

### PAGE and Western blotting

Samples were separated on 8% Tris–HCl SDS-polyacrylamide gels (BioRad, Berkeley, USA) and transferred to C membranes (Amersham). After transfer, filters were blocked for 1h at room temperature using PBS containing 0.1% v/v Tween-20 (PBS-T), 5% w/v milk powder. The anti-FLAG (FG4R) and anti-His (HIS.H8) antibodies were used at 1μg/ml in PBS-T, 3% w/v milk powder. Following several washes with PBS-T, membranes were incubated for 1h with an anti-mouse HRP-conjugated secondary antibody and the bound antibodies detected by SuperSignal West Pico chemiluminescence substrate (Thermo Fisher Scientific).

### Cells and cell transfection

HEK293T cells were kindly provided by Dr Claudine Porta (University of Oxford—obtained October 2014) (source: ATCC, CRL-3216). Cells were maintained in Dulbecco’s modified Eagle’s medium (DMEM) supplemented with 10% (v/v) heat inactivated foetal calf serum (FCS), 2mM L-glutamine, 100U/ml penicillin and 100μg/ml streptomycin. Cells were grown in 6-well plates until 80% confluence and transfected with 2.5μg plasmid DNA per well using Lipofectamine 2000 (Life-Technologies/Thermo Fisher Scientific) at a ratio of 1:6 DNA to Lipofectamine. Plasmid DNA and Lipofectamine were added separately to Opti-mem (Life-Technologies/ Thermo Fisher Scientific) and left to incubate at room temperature for 5mins. The DNA and Lipofectamine solutions were then mixed (transfection solution) and left at room temperature for 20mins. The media was removed and the cells washed with PBS. The transfection solution was then added to each well and incubated at 37°C for 5h. Cells were then supplemented with media with 10% FCS. After 24h, the cells were washed and incubated in fresh cell culture media containing 1% FCS. Cells were incubated for a further 48h at 37°C before harvesting the supernatant. Supernatants were clarified and stored for downstream analysis.

### Affinity purification

Truncated FLAG tagged αvβ6 was purified using an anti-FLAG M2 affinity gel (Sigma). Briefly, the affinity gel was equilibrated with Tris buffered saline (TBS: 50mM Tris pH7.6, 150mM NaCl) and cell culture supernatant (containing FLAG-tagged αvβ6) added with continuous mixing for 3h at room temperature. The slurry was loaded onto a spin column and the resin washed with 10x column volumes of TBS. The integrin was eluted by competition with a 3xFLAG peptide (Sigma Aldrich) 100μg/ml in TBS, in batches of 1 column volume.

Truncated His-tagged αvβ6 was purified using a His-Trap FF column (GE Healthcare) pre-packed with 1ml Ni-sepharose. Solutions were passed through the column using a peristaltic pump (flow rate 1ml/min). The column was first washed with 5x column volumes of water, followed by 5x column volumes of 500mM imidazole elution buffer (20mM Sodium phosphate dibasic, 0.5M NaCl, 500mM Imidazole), and finally 10x column volumes of binding buffer (20mM Sodium phosphate dibasic, 0.5M NaCl, 10mM Imidazole). Imidazole and NaCl were added to cell culture supernatant containing his-tagged αvβ6 to final concentrations of 10mM and 500mM respectively, and the supernatant passed through the column. The column was washed with 5x column volumes of binding buffer. The integrin was then eluted by the sequential addition of 2x column volumes of elution buffers (20mM Sodium phosphate dibasic, 0.5M NaCl) containing increasing concentrations of imidazole (elution buffers 1–6 contained 15.6mM, 31.25mM, 62.5mM, 125mM, 250mM and 500mM imidazole respectively).

### Antigen detection ELISA methods

#### Using immobilised integrin for detection of empty capsids

Plastic 96-well plates (Nunc Maxisorp immunoplates) were coated with FLAG-tagged, affinity purified, truncated αvβ6 (~10μg/ml) in TBS supplemented with 2 mM CaCl_2_ and 1 mM MgCl_2_ (TBScm) or with 100μl of undiluted FLAG-tagged, truncated αvβ6 (as transfected cell supernatants) overnight at 4°C. The wells were washed with TBScm containing 0.05% tween before adding 100μl (1μg/ml) of purified FMDV EC in binding buffer (TBScm with 2% bovine serum albumin) and incubating for 1h at room temperature. The wells were washed three times with TBScm containing 0.05% tween and bound EC were detected by sequential incubation with a guinea pig, anti-A22 Iraq polyclonal antisera used at 1/1000 followed by a rabbit, anti-guinea pig horseradish peroxidase (HRP) conjugated secondary antibody (Sigma) used at 1/5000 for 45min each at room temperature. After each incubation, the wells were washed three times with TBScm containing 0.05% tween. HRP substrate (OPD: Sigma) was added and the optical density of the wells was read at 450nm after 0.5h. Competing peptides (1μM) were mixed with the EC (1μg/ml) prior to addition to the wells. Similarly, EDTA (1mM) was added to the EC before addition to the wells. Alternatively, wells coated with integrin supernatants were incubated directly with the anti-FLAG antibody or MAb 10D5 (specific for the αvβ6 heterodimer) both used at 1/1000 and bound antibodies were detected using HRP conjugated secondary antibodies and the OD determined at 450nm as above.

#### Using immobilised rabbit polyclonal sera to detect empty capsids

The assay was carried out as described above except that EC were trapped using a rabbit, anti-A22 Iraq polyclonal sera (1/1000) in place of the integrin.

#### Using immobilised integrin for detection of FMDV

The assay was carried out similar to the protocol described in Ferris et al [17] for the indirect sandwich ELISA using polyclonal antisera as the capture reagent, with the exception that the plates were coated with 100μl of undiluted FLAG-tagged (as transfected cell supernatant) or His-tagged (purified ~1μg/ml), truncated αvβ6 cell culture supernatant and the development time was 15–25mins. FMDV infected cell lysates and homologous serotype-specific guinea pig antiserum or mouse monoclonal antibodies were used in combination with the appropriate secondary antibodies (rabbit anti-guinea pig or rabbit anti-mouse conjugated to HRP) to detect bound viruses.

#### Using the integrin to detect immobilised empty capsids

Plastic 96-well plates were coated with 100μl (1μg/ml) of purified EC overnight at 4°C. The coated wells were washed with TBScm and incubated with undiluted integrin supernatants (100μl) for 1h at room temperature. The wells were washed three times with TBScm and incubated sequentially with the anti-FLAG antibody and a mouse horseradish peroxidase (HRP) conjugated secondary antibody (Sigma) for 45 min each at room temperature with washing between antibodies. Wells were washed three times with TBS and HRP substrate (OPD: Sigma) was added. The optical density of the wells was read at 450nm.

### Viruses and antibodies

BEI-inactivated cell culture supernatants (Reference FMDV antigens) were obtained from the FMD World Reference Laboratory (WRLFMD) at The Pirbright Institute, and were prepared using the following viruses: O1BFS 1860 (FMDV Type O), A22 IRQ (FMDV Type A), C3 PHI (FMDV Type C), BOT 1/68 (FMDV SAT 1), ZIM 5/81 (FMDV SAT 2), ZIM 4/81 (FMDV SAT 3), CAM 9/80 (Asia1) and UKG 27/72 (swine vesicular disease virus, [SVDV]). The following FMDV field isolates, SAT 3 KNP 48/1991 (PK1 RS3 BTY1 BHK1), A TAN 15/2013 (BTY2), C BHU 10/1991 (BTY2 BHK1), SAT 1 KEN 4/2013 (BTY2 BHK1), ASIA 1 TUR 23/2014 (BTY2 BHK1), O IRN 72/2009 (BTY2 BHK1) and SAT2 ZIM 1/2014 (BTY2 BHK1) were also from the WRLFMD and were used as untreated, infected-cell culture supernatants. The passage history of each virus is given in parenthesis. PK: Primary pig Kidney cells; RS: IBRS2 cells; BTY: Primary bovine thyroid cells; BHK: Baby hamster kidney cells.

All viruses were used at a dilution that has been previously shown to allow detection by a conventional FMDV ELISA using polyclonal antibodies to trap virus. SVDV was included in ELISA experiments as it is used as part of the routine diagnostics in the WRLFMD.

The guinea pig and rabbit anti-A22 Iraq polyclonal sera and the other serotype-specific guinea pig polyclonal sera were also obtained from the WRLFMD. The following guinea pig polyclonal sera were used; O1 BFS 1860 Gpig B 1/10 P3 26/3/84 (FMDV Type O), A5/22/24 Comb Gpig B 1/10 S2 13/2/97 (FMDV Type A), C3 Resende Gpig B 1/10 D1 13/11/96 (FMDV Type C), BOT 1/68 Gpig B 1/10 29/11/93 (FMDV Type SAT1), ZIM 5/81 Gpig D 1/10 26/10/94 (FMDV Type SAT2), ZIM 4/81 Gpig B 1/10 S1 16/11/93 (FMDV Type SAT3), CAM 9/80 Gpig B 1/10 S 7/10/05 (FMDV Type Asai-1), and SVDV GP-a-146s GP D 1/10 3/8/92 (SVDV).

The FMDV MAbs were as follows; type-A, A 5F6 and A 4D12 raised against FMDV A24 Cruzeiro and A Iran 96 respectively; type-O, O 7E1, O 3C8, O 3B11 raised against O1 Italy 93, O1 Lausanne and O1 Manisa respectively; type-C, C 3E9 raised against C Brescia 64, Asia-1, ASIA 1 3D8 raised against NEP 29/1997, SAT1, SAT 1 HD7 raised against KEN 11/2005, SAT2, SAT 2 2H6 raised against ZIM 5/1981, and, SAT3, SAT 3 C14, SAT 3 BH5 and SAT 3 FE11 all raised against ZIM 4/1981 [18]. The MAbs were not purified and were used at a dilution that had previously been shown to detect FMDV in validated diagnostic ELISA assays.

The anti-FLAG antibody (FGR4: MA1-91878) was from Thermo Fisher Scientific, the anti-6X His tag^®^ antibody [HIS.H8] (ab18184) from Abcam, and the MAb to αvβ6 (MAb 10D5) was from Millipore. Rabbit anti-guinea pig immunoglobulins and rabbit anti-mouse immunoglobulins conjugated to HRP were from Dako.

### Peptides and reagents

The FMDV 17-mer peptide (VPNLRGDLQVLAQKVAR) and the control RGE version were synthesized by Peptide Protein Research (Bishops Waltham, Southampton, United Kingdom). The sequence of this peptide was derived from the VP1, GH loop of FMDV O1Kcad2. Truncated human αvβ6 was from R&D Systems. EDTA was from Sigma and HRP substrate from Thermo Fisher Scientific.

## Results and Discussion

### Production of FMDV A22 Iraq empty capsids

**Fig 1:**
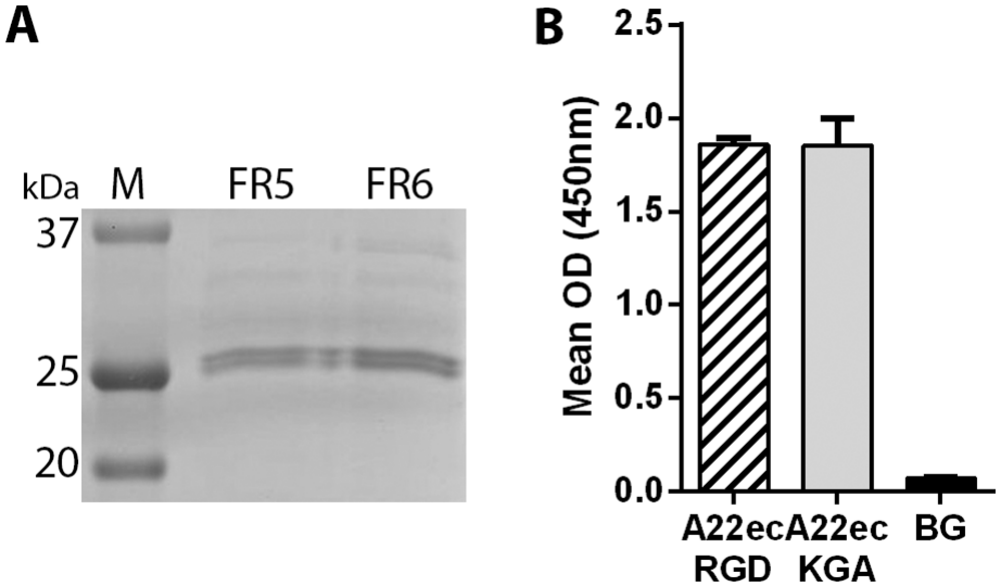
Characterisation of FMDV empty capsids. (A) 12% PAGE for fractions (FR5 and FR6) collected from a sucrose gradient for purification of stabilised FMDV EC (A22ecKGA). The major bands represent VP1-3. VP1 runs slightly slower than VP2 and VP3, which co-migrate as a single band. M = molecular weight markers. (B) Sandwich ELISA for detection of FMDV EC (A22ecRGD and A22ecKGA) captured using a rabbit, anti-A22 Iraq polyclonal sera and detected using a guinea pig anti-A22 Iraq polyclonal sera. BG (Background) shows the optical density (OD) for the assay carried out in the absence of EC.

FMDV EC (also known as virus-like particles) can be produced using a vaccinia virus expression system and readily purified on sucrose gradients [19, 20]. In addition, recombinant FMDV and EC have been produced with mutations that result in stabilised capsids that are resistant to fluctuations in pH and temperature [19, 21–24]. Previously, these approaches have been used to produce and characterise (by electron microscopy and antigenic profiling) EC for FMDV A22 Iraq, which have stabilising disulphide bonds across the pentamer-pentamer interfaces [19, 20]. FMDV binding to αvβ6 is dependent on a conserved RGD motif on the G-H loop of VP1 (see Introduction). To demonstrate FMDV binding to truncated αvβ6 preparations (see below), we produced matched preparations of stabilised FMDV A22 Iraq EC with either the wild-type RGD sequence (A22ec/RGD) (see reference 19) or a biologically inactive KGA (A22ec/KGA) in place of the RGD (Fig 1A). The RGD to KGA change will abrogate integrin binding and also would be expected to reduce antibody binding as the G-H loop of VP1 forms an independent antigenic site (Site 1) on the capsid [25–28]. However, this change would not be expected to alter capsid stability or recognition of other major antigenic sites on the capsid (sites 2, 3 and 4). When used in a standard sandwich ELISA, the purified A22 Iraq EC were readily detected when trapped by an anti-A22 Iraq rabbit polyclonal sera and detected using anti-A22 Iraq guinea-pig polyclonal sera (Fig 1B). This demonstrates that the KGA EC retain antigenicity, despite having mutations at the RGD, and can be used to confirm RGD-dependent interactions with integrins (see below).

### Expression and purification of functional, truncated, FLAG- or His-tagged bovine integrin αvβ6

**Fig 2:**
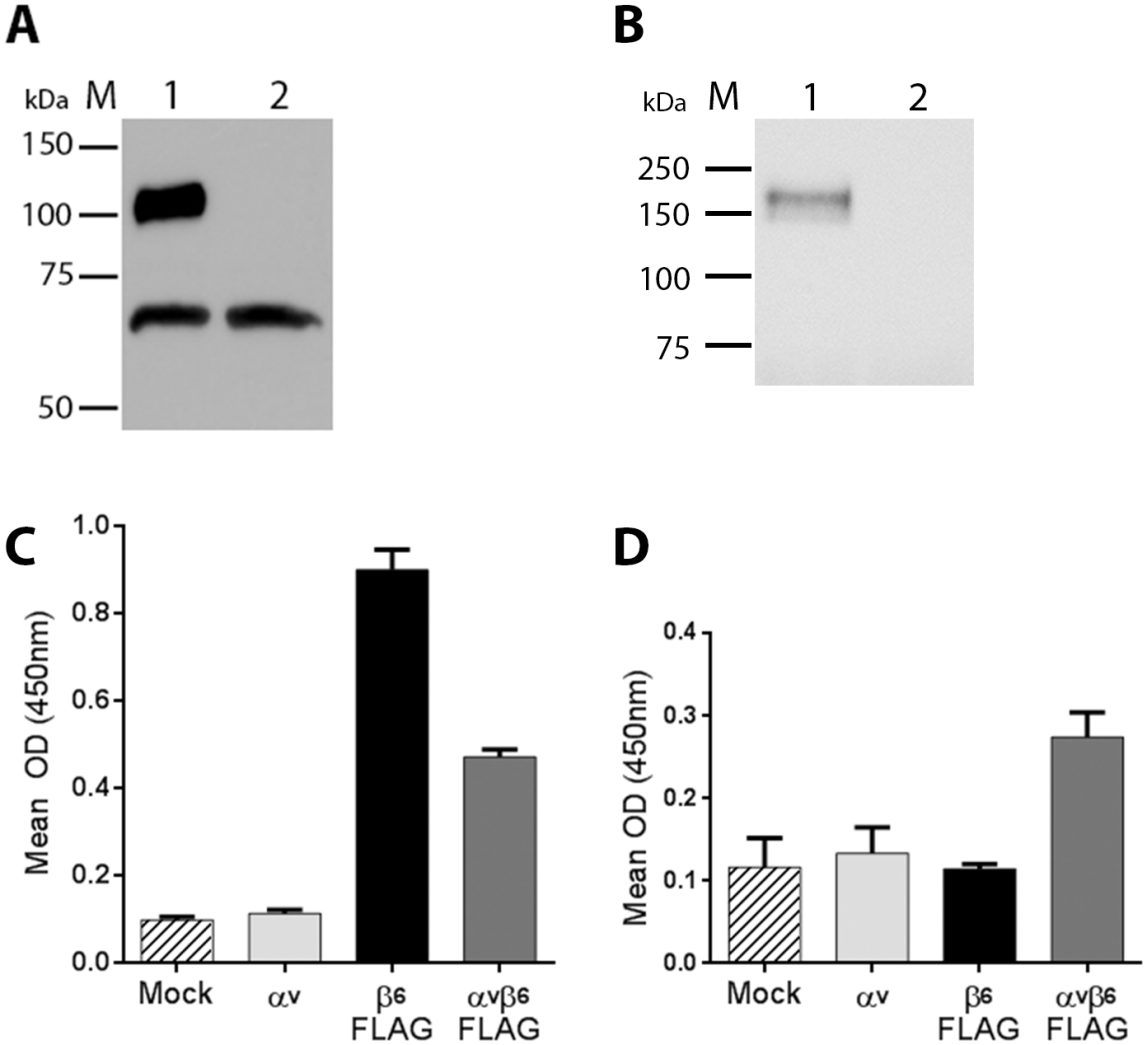
Expression of truncated, FLAG- or His-tagged bovine αvβ6. (A) Western blot of cell culture supernatants for αvβ6-FLAG transfected (Lane 1) or mock-transfected (Lane 2) cells using an anti-FLAG antibody. The upper band (Lane 1) corresponds to the FLAG-tagged β6 subunit. (B) A Western blot of cell culture supernatants for αv-HIS/β6 transfected (Lane 1) or mock-transfected (Lane 2) cells using an anti-His antibody. The band (Lane 1) corresponds to the His-tagged αv subunit. M = molecular weight markers. (C) and (D) ELISA for integrin expression. Wells were coated with cell culture supernatants for mock-transfected cells (Mock), cells transfected with expression plasmids for the αv (non-tagged) or β6-FLAG subunits individually, or co-transfected with both αv and β6-FLAG plasmids. The detecting antibody used in panel (C) was anti-FLAG and in panel (D) an antibody specific for the αvβ6 heterodimers (MAb 10D5).

HEK293T cells were transiently co-transfected (αvβ6-transfected) with αv (non-tagged) and β6-FLAG expression plasmids, or mock-transfected and transfected cell supernatants probed for the FLAG epitope at 72h post-transfection (see Methods). A band corresponding in size to the truncated, FLAG-tagged β6 subunit was detected in the αvβ6-transfected cells (Fig 2A, lane 1). A lower non-specific band was also detected with the anti-FLAG antibody in both the mock (lane 2) and αvβ6-transfected cell supernatants. Fig 2B shows a similar experiment where cells were mock-transfected or co-transfected with His-tagged αv and non-tagged β6 and probed for the His tag. A single band corresponding to the truncated His-tagged αv subunit was only detected in the co-transfected cells (Lane 1) but not in the control, mock-transfected cells (Lane 2).

To further demonstrate integrin expression, ELISA plates were coated with supernatant from mock-transfected cells, cells co-transfected with both αv and β6-FLAG plasmids (αvβ6-FLAG) or cells transfected with the αv or β6-FLAG plasmids individually and then probed in an ELISA using the anti-FLAG antibody. A positive signal was detected for the αvβ6-FLAG and the β6-FLAG transfected cells (Fig 2C), but not for the mock or αv transfected cells (Fig 2C). The higher signal for the supernatants from the cells transfected with only the β6-FLAG plasmid may be due to secretion or leakage of the β6-FLAG subunit into the cell culture media when over expressed on its own. However, when the ELISA was repeated using MAb 10D5 (Fig 2D) only cells co-transfected with both αv and β6-FLAG gave a positive signal. As MAb 10D5 recognises the RGD-binding site and is specific for the αvβ6 heterodimer these results indicate that the αv and β6-FLAG subunits are correctly associated and secreted into the cell supernatant.

**Fig 3:**
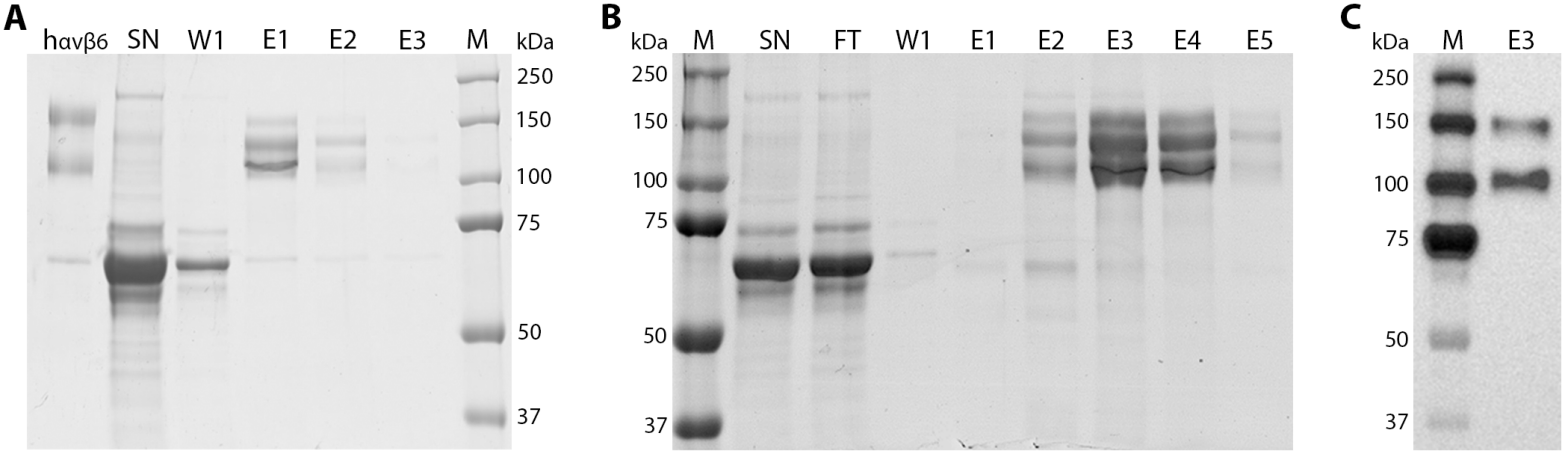
Purification of truncated, FLAG- or His-tagged bovine αvβ6. (A) PAGE analysis of purified bovine αvβ6-FLAG. Lanes represent: crude cell culture supernatant (SN), the first wash step (W1), successive elution steps (E1–E3), and 2μg truncated human αvβ6 (h αvβ6). (B) PAGE analysis of purified, bovine αv-His/β6-His. Lanes represent: crude cell culture supernatant (SN), column flow through (FT), the first wash step (W1), and successive elution steps (E1–E5). (C) Western blot of αv-His/β6-His elution three (E3) using the anti-His antibody. M = molecular weight markers.

The truncated, αvβ6-FLAG integrin was purified using anti-FLAG beads, as per manufacturer recommendations. Most of the αvβ6 integrin was eluted from the beads in the first elution step (Fig 3A, lane E1). However, three bands were seen on polyacrylamide gel electrophoresis (PAGE) of the samples. The upper and lower bands are the expected molecular weight for the αv and FLAG-tagged β6 subunits respectively and migrated to a similar position to truncated human αvβ6.

To identify the αv and β6 subunits, His-tagged αvβ6 protein (tagged on both subunits) was also purified using a column packed with nickel-sepharose beads (His Trap FF–GE Healthcare), and eluted from the column by sequential elutions using increasing concentrations of imidazole, as described in Materials and Methods. Most of the αvβ6 integrin was eluted from the beads in the third elution step (Fig 3B, lane E3). Again, three bands were seen on PAGE analysis and the upper and lower bands correspond in size to the His-tagged αv and β6 subunits respectively. To confirm this, the third elution sample was probed using an anti-His antibody (Fig 3C). Only two bands were seen that correspond to the upper and lower bands on the PAGE analysis indicating that the middle band did not include His tagged proteins. The identity of the middle band is currently not known and will need further investigation but it could arise as a further truncation of the αv subunit that removes the C-terminal His tag during transfection or during purification.

### Authentic RGD- and divalent cation-dependent FMDV EC binding to truncated soluble FLAG-tagged bovine integrin αvβ6

**Fig 4:**
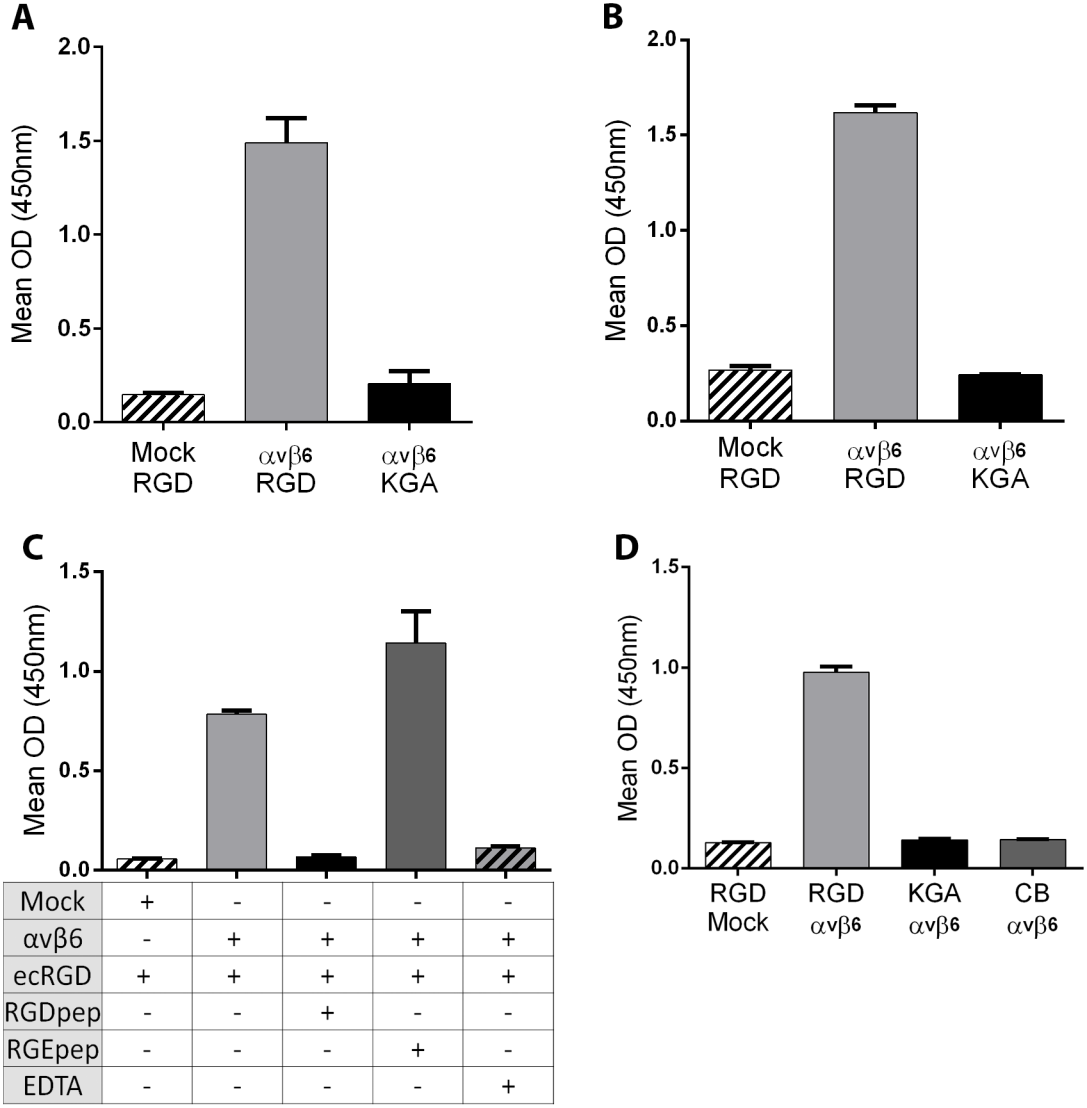
Authentic RGD- and divalent cation-dependent binding of FMDV empty capsids to truncated, FLAG-tagged bovine αvβ6. (A) Sandwich ELISA for the detection of FMDV EC. Wells were coated with cell culture supernatant from mock-transfected cells (Mock) or cells co-transfected with expression plasmids for the αv and β6-FLAG subunits (αvβ6) and incubated with FMDV A22ecRGD (RGD) or A22ecKGA (KGA). FMDV capsid proteins were detected using an anti-A22 Iraq, guinea-pig polyclonal sera. (B) The same ELISA using purified αvβ6-FLAG in place of transfected cell supernatants. (C) Wells were coated with cell culture supernatant from mock-transfected cells (Mock) or cells co-transfected with both αv and β6-FLAG subunits (αvβ6) and incubated with FMDV A22ecRGD (ecRGD) in the presence or absence of an RGD-containing peptide, an inactive control RGE peptide or EDTA (as indicated on the figure). (D) An ELISA using truncated αvβ6-FLAG to detect FMDV empty capsid. Wells were coated with A22ecRGD (RGD), A22ecKGA (KGA) or coating buffer alone (CB) and capsid proteins detected using FLAG-tagged truncated αvβ6 (αvβ6) i.e. cell culture supernatant from αvβ6 co-transfected cells. RGD/Mock wells were coated with A22ecRGD and incubated with cell culture supernatant from mock-transfected cells.

As stated above, FMDV binding to αvβ6 is dependent on the VP1 RGD [14]; however, binding is also dependent on divalent cations [13]. An ELISA detected a positive signal for A22ecRGD (RGD) in the wells coated with co-transfected cell-supernatant (αvβ6-FLAG) but not in wells coated with mock-transfected cell supernatant (Fig 4A). In addition, a signal was not detected for the wells coated with co-transfected cell-supernatant (αvβ6-FLAG) and incubated with A22ecKGA (KGA) thus confirming that the binding of EC to αvβ6 is RGD dependent. Similar results were observed using purified FLAG-tagged, truncated αvβ6 (Fig 4B) to trap the EC, as a positive signal was only detected for A22ecRGD. Additionally, FMDV A22ecRGD binding to αvβ6 integrin was inhibited by a synthetic RGD-containing peptide with its sequence derived from the VP1 G-H loop of FMDV O1Kcad2 (but not by a control inactive RGE peptide) or by EDTA (Fig 4C). These results demonstrate that binding of FMDV EC to truncated FLAG-tagged bovine αvβ6 is dependent on both the FMDV RGD and divalent cations. Bovine αvβ6 can also be used as part of the detector system (rather than a trapping reagent), in that a positive signal was only seen for the wells coated with A22ecRGD (but not for A22ecKGA) when a combination of the FLAG-tagged integrin (i.e. cell culture supernatant from αv and β6-FLAG co-transfected cells) and an anti-FLAG antibody were used for detection (Fig 4D).

#### Soluble truncated, bovine integrin αvβ6 binds all FMDV serotypes

**Fig 5:**
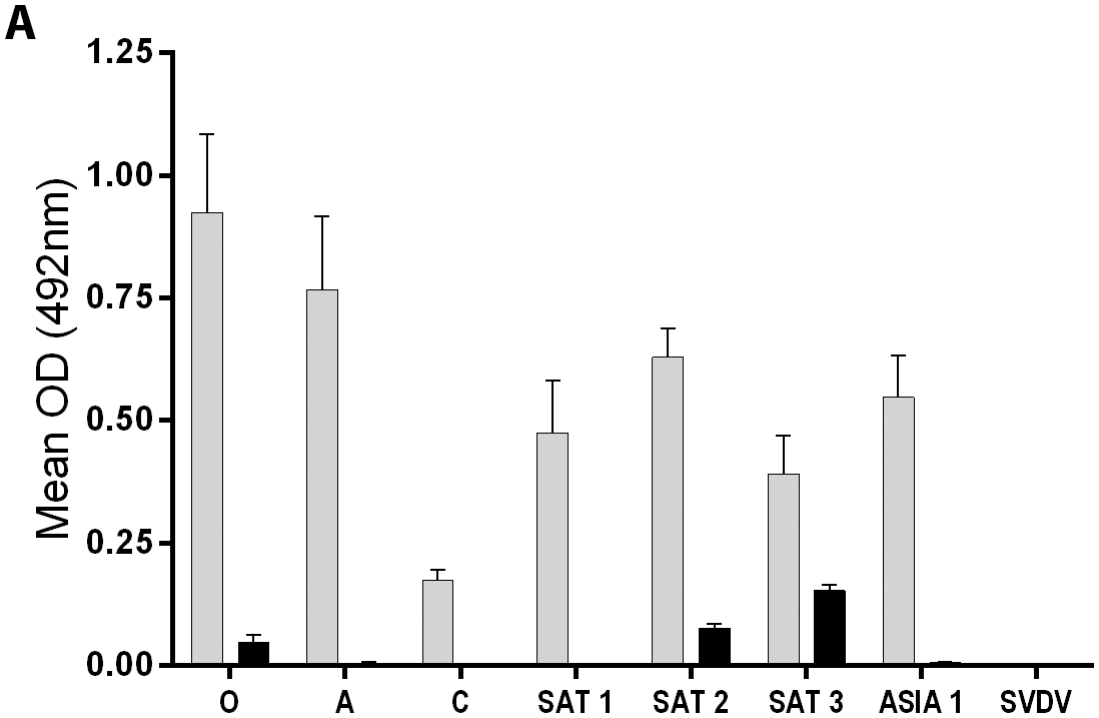
Detection of FMDV serotypes using truncated, FLAG-tagged bovine αvβ6 with anti-FMDV polyclonal sera. Wells were coated with cell culture supernatants from mock-transfected cells (black bars) or cells co-transfected with expression plasmids for the αv and β6-FLAG subunits (grey bars), and incubated with viruses representative of the different FMDV serotypes (as indicated) or with SVDV, and detected using FMDV serotype-specific or SVDV-specific polyclonal antibodies raised in guinea pigs.

FMDV exists as seven antigenically distinct serotypes; hence, virus serotyping requires seven different serotype-specific trapping rabbit antisera. However, as demonstrated here, the truncated αvβ6-FLAG can be used to detect viruses representative of all FMDV serotypes (Fig 5), but not SVDV. The lower signal with the polyclonal antibody to type C FMDV (Fig 5) could be due to a lower binding affinity of type C virus to bovine αvβ6 or a poor match with the detecting sera.

**Fig 6:**
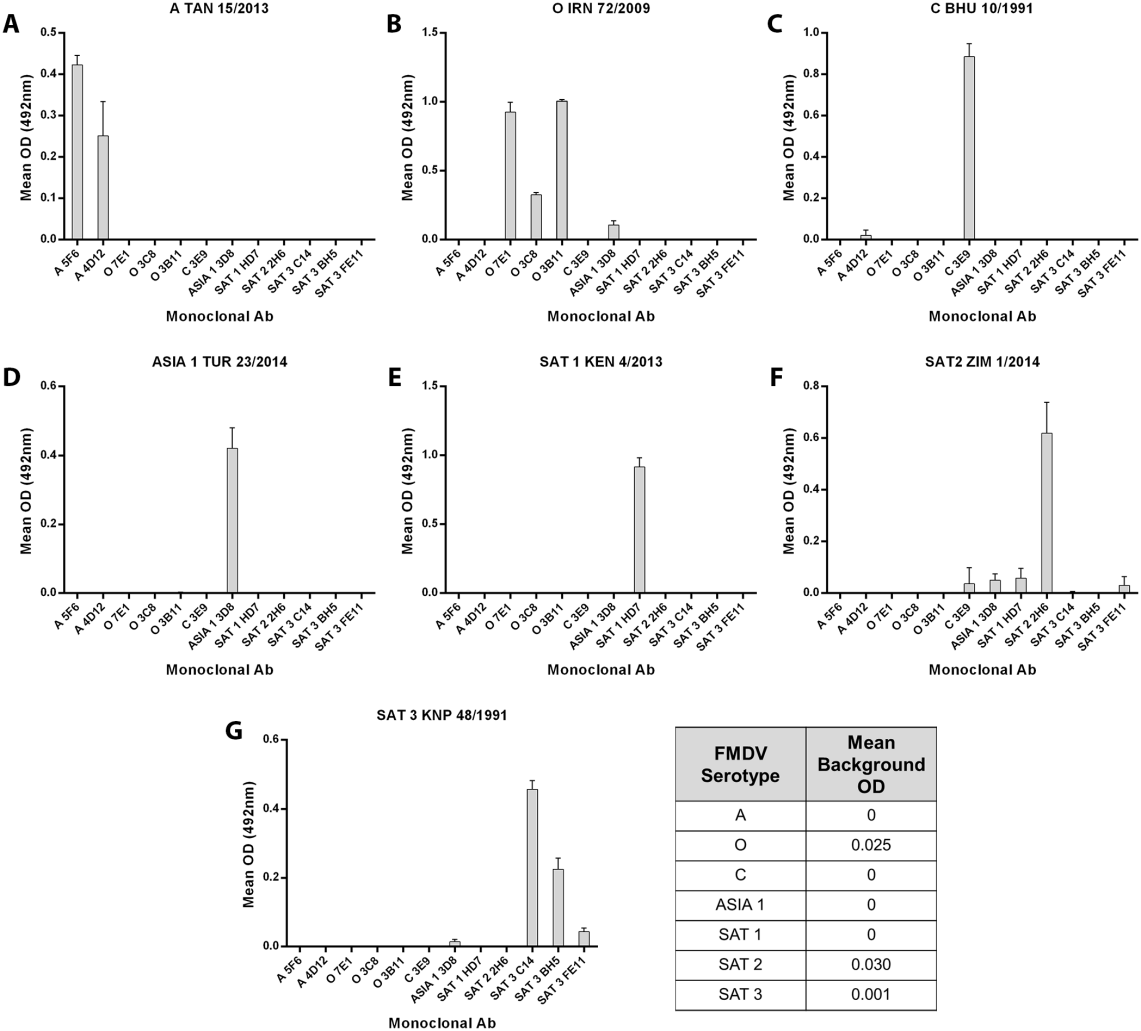
Detection of FMDV serotypes using truncated, His-tagged bovine αvβ6 with anti-FMDV monoclonal antibodies. Wells were coated with purified, His-tagged αvβ6 and incubated with viruses representative of the different FMDV serotypes (Panels A-G as indicated). FMDV was detected using a range of FMDV serotype-specific MAbs (see methods for antibody details). The background OD for each MAb was determined by excluding the virus from the assay. The background OD was then subtracted from the OD values obtained for viruses. The mean background for each MAb is shown on the figure.

A similar experiment was carried out using purified HIS-tagged αvβ6 and a range of FMDV field isolates (including more recent outbreak strains) with anti-FMDV, serotype-specific Mab’s for detection (Fig 6). Each virus gave a strong positive signal (including for type C FMDV) only when detected with the appropriately matched MAb, and little or no cross-serotype reactivity of the MAbs was seen. Together, the results shown in Figs 5 and 6 demonstrate the potential of the truncated, bovine αvβ6 integrin as a trapping reagent for FMDV samples regardless of serotype, and when combined with either polyclonal anti-sera or with MAbs; however, further experiments are needed in order to optimise and validate the assay for routine diagnosis of FMDV.

Preparedness through virus surveillance and response activities, such as early diagnosis, are essential for effective control of FMD. Many surveillance and diagnostic assays are dependent on high-affinity, anti-FMDV serotype-specific antibodies in small animals (rabbits and guinea pigs) that give broad virus coverage, including to new emerging strains. This necessitates the periodic need to generate new antisera and testing for reactivity to new strains. We have shown previously that FMDV is highly adapted to use an integrin αvβ6 as a receptor to initiate infection [17]. Here we show that truncated, bovine integrin αvβ6 can be produced by transient transfection of mammalian cells and used as a universal trapping reagent for the detection of all FMDV serotypes in combination with detecting FMDV-specific polyclonal sera or MAbs. Furthermore, given that the VP1 RGD sequence, and the use of integrin receptors are highly conserved features of FMDV, it is expected that integrin could be used to trap new emerging strains. In addition, we also show that non-infectious, stabilised, FMDV EC can be used to replace FMDV antigen as a positive control in diagnostic assay. This could negate the need to produce large amounts of infectious virus, under high containment conditions, for use as a positive control. In conclusion, the use of αvβ6 and EC could reduce the need for raising polyclonal sera and overcome the need to produce inactivated positive control viruses, as well as provide more reliable FMD diagnosis.
